# Comparison of multiphase data from CT perfusion vs clinical 4-phase CT scans with respect to image quality, lesion detection, and LI-RADS classification in HCC patients

**DOI:** 10.1016/j.heliyon.2022.e08757

**Published:** 2022-01-13

**Authors:** A. Mohammadi, W. Bartholmae, M. Woisetschläger

**Affiliations:** a Department of Radiology, Department of Medical and Health Sciences, Linköping, Sweden; b Center for Medical Image Science and Visualization (CMIV), Linköping University, Linköping, Sweden

**Keywords:** Perfusion imaging, HCC, 4-Dimensional computed tomography, Image quality, CT

## Abstract

**Purpose:**

The aim of this study was to assess the image quality and diagnostic performance of reconstructed arterial (A) and portal venous (PV) phases in computed tomography perfusion (CTP) scans compared to the corresponding phases in standard 4-phase CT and to assess the utility for LI-RADS classification using CTP and 4-phase 4CT.

**Methods:**

A total of 26 scans with each method (CTP and 4-phase CT) from 19 hepatocellular carcinoma patients were analyzed and compared. Arterial and PV phases reconstructed by advanced modeled iterative reconstruction at strength 4 (ADMIRE 4) from raw CTP data were compared with image sets from arterial and PV phases of 4-phase CT (ADMIRE 3) in the same patient with respect to image quality.

**Results:**

Quantitative image analysis showed that reconstructed CTP datasets were equivalent to 4-phase CT image sets. Qualitative image analysis revealed similar lesion detection rates with the 2 methods for patients with an abdominal diameter ≤36 cm and body weight <90 kg, but lower detection rates with CTP for patients with an abdominal diameter >37 cm. There was no difference in Liver Imaging Reporting and Data System (LI-RADS) classifications between the 2 methods.

**Conclusion:**

Reconstructed CTP images can potentially replace 4-phase CT images in patients weighing <90 kg and with a body diameter <37 cm, as the 2 methods are comparable in terms of quantitative image quality and ability to detect and classify lesions based on LI-RADS criteria.

## Highlights

• Reconstructed A- and PV-CTP images have comparable image quality to 4-phase CT.
• Reconstructed A- and PV-CTP images can be used for LI-RADS classification of HCC.
• A-/PV-CTP has the potential to reliably detect lesions in patients weighing <90 kg with body diameter ≤36 cm.

## 1 Introduction

Accurate early detection of hepatocellular carcinoma (HCC) and its differentiation from benign tumors is critical for achieving optimal treatment outcomes. Four-phase computed tomography (CT) of the liver and multiparametric magnetic resonance imaging (MRI) are the gold-standard imaging modalities recommended by the European Association for the Study of the Liver and American Association for the Study of Liver Disease for the diagnosis of liver tumors [1, 2, 3, 4]. In 4-phase CT, bolus tracking yields reproducible images of non-contrast, arterial (A), portal venous (PV), and delayed (D)PV phases [5]. However, this method uses a fixed scan delay after contrast enhancement, which can lead to suboptimal arterial phase enhancement because of interpatient variations in age, body weight, cardiac output, and kidney function [6]. As such, tumors might still not be optimally enhancing in the predefined arterial phase given that arterial phase enhancement and correct phase timing are essential for Liver Imaging Reporting and Data System (LI-RADS) assessment. This may lead to difficulties in the interpretation of CT images where the tumor arterial enhancement pattern shows small variations [7].

Furthermore, assessment of treatment effects of systemic chemotherapy can be challenging when there is no change in tumor size [8]. LI-RADS assessment of the response to interventional radiologic treatments such as transarterial chemoembolization (TACE) or ablation depends on high-quality arterial phase and portalvenous phase images [9]. Therefore, new imaging or image analysis techniques are needed that maximize detection rates and obtain accurate and quantitative information on treatment response and associated adverse effects.

Computed tomography perfusion (CTP) is an alternative imaging method to 4-phase CT in which scans are repeatedly performed over the same region of interest (ROI), including the whole upper abdomen, yielding image stacks at different time points starting with native images (images without contrast) and several high temporal resolution series in the early and late arterial phases, as well as series with lower temporal resolution from the late arterial phase to the PV phase [8]. In this manner, the problem of missing the point of optimal arterial enhancement of the liver or lesions of interest that is associated with 4-phase CT can be avoided. It was demonstrated that CTP has a high sensitivity for detecting liver lesions (94%) and is also appropriate for tumor diagnosis and anticancer treatment response monitoring [10]. Additionally, CTP provides quantitative imaging data on PV and arterial components of hepatic blood flow that can facilitate HCC diagnosis [1, 2]; additionally, quantitative parameters in CTP were shown to be related to high vessel density in tumors [11, 12].

Recent technical advances including wide-detector CT and new image reconstruction and motion correction algorithms have enhanced the diagnostic utility of CTP [8]. However, there are certain limitations to this method such as respiratory motion artifacts and reduced image quality per collected dataset as the subject is scanned several times with a lower radiation dose at each time point. Moreover, reviewing thousands of CT images is time-consuming and can increase the risk of overdiagnosis [13, 14]. It is therefore essential to develop an image reconstruction method that yields high-quality images with maximum diagnostic information that are amenable to interpretation. In previous investigations with time average images in perfusion scans, CTP images were only reconstructed by filtered back projection [14, 15], unlike the iterative reconstruction kernels used in the present work. Additionally, these studies used a high radiation dose, which was reduced in our study through a combination of low kilovoltage peak (kVp) CT and an iterative reconstruction algorithm.

The purpose of this study was to compare the image quality, diagnostic performance, and utility for LI-RADS classification of reconstructed arterial and PV phases of liver CTP examinations with the corresponding phases in standard 4-phase CT images in order to determine whether CTP can replace 4-phase CT as a diagnostic method for HCC.

## 2 Materials and methods

### 2.1 Patients

A total of 19 HCC patients (14 men and 5 women) with a mean age ±standard deviation (SD) of 70 ± 8 years (range: 55–86 years), mean weight of 90 ± 22 kg (range: 60–130 kg), and mean abdominal diameter of 37 ± 4.8 cm (range: 31–50.6 cm) underwent CT examinations, including a clinical 4-phase CT of the abdomen and CTP of the liver during the same visit at Linköping University Hospital between October 2016 and March 2019. The examinations were performed the day before TACE treatment. Seven patients underwent follow-up examinations 3 weeks after TACE with the same protocol. In total, 26 examinations with each method were included in the analysis. Inclusion criteria were HCC patients undergoing TACE who were able to provide informed consent and had normal kidney function. Abdominal diameter was measured on the axial CT scan at the level of the second lumbar vertebra in the horizontal plane.

### 2.2 Liver CTP protocol

The study was performed using a 192-row CT scanner (FORCE dual source; Siemens, Munich, Germany). The tube voltage was 70 kVp and the tube current was 150 mAs with a collimation of 48 × 1.2 or 192 × 0.6 for a total of 25 scans. The scans were performed during free shallow breathing; the scan duration was 45.45 s (contrast delay ca 52 s) with a scan length of 22.5 cm over the upper abdomen. The first scan was initiated 7 s after injection of low-osmolarity nonionic contrast medium (Iopromid, Ultravist 370 mg I/ml; Bayer Healthcare, Leverkusen, Germany; 50-ml fixed dose at an injection speed of 6 ml/s). The first 20 scans were taken every 1.5 s and the last 5 were taken every 3 s.

### 2.3 Liver 4-phase CT protocol

The same CT scanner as for the CTP protocol was used to perform a 4-phase CT scan (regarding our clinical routine) with a tube voltage of 120 kVp and tube current 130 mAs. The examination included a non-enhanced scan and 3 contrast-enhanced phases—namely, arterial, PV, and delayed venous phases. Low-osmolarity nonionic contrast medium was injected at a maximum volume of 118 ml and rate of 5–6 ml/s. Scans of arterial, PV, and DPV phases were acquired 25 s, 60 s, and 4 min, respectively, after bolus injection.

### 2.4 Image reconstruction

**Figure 1:**
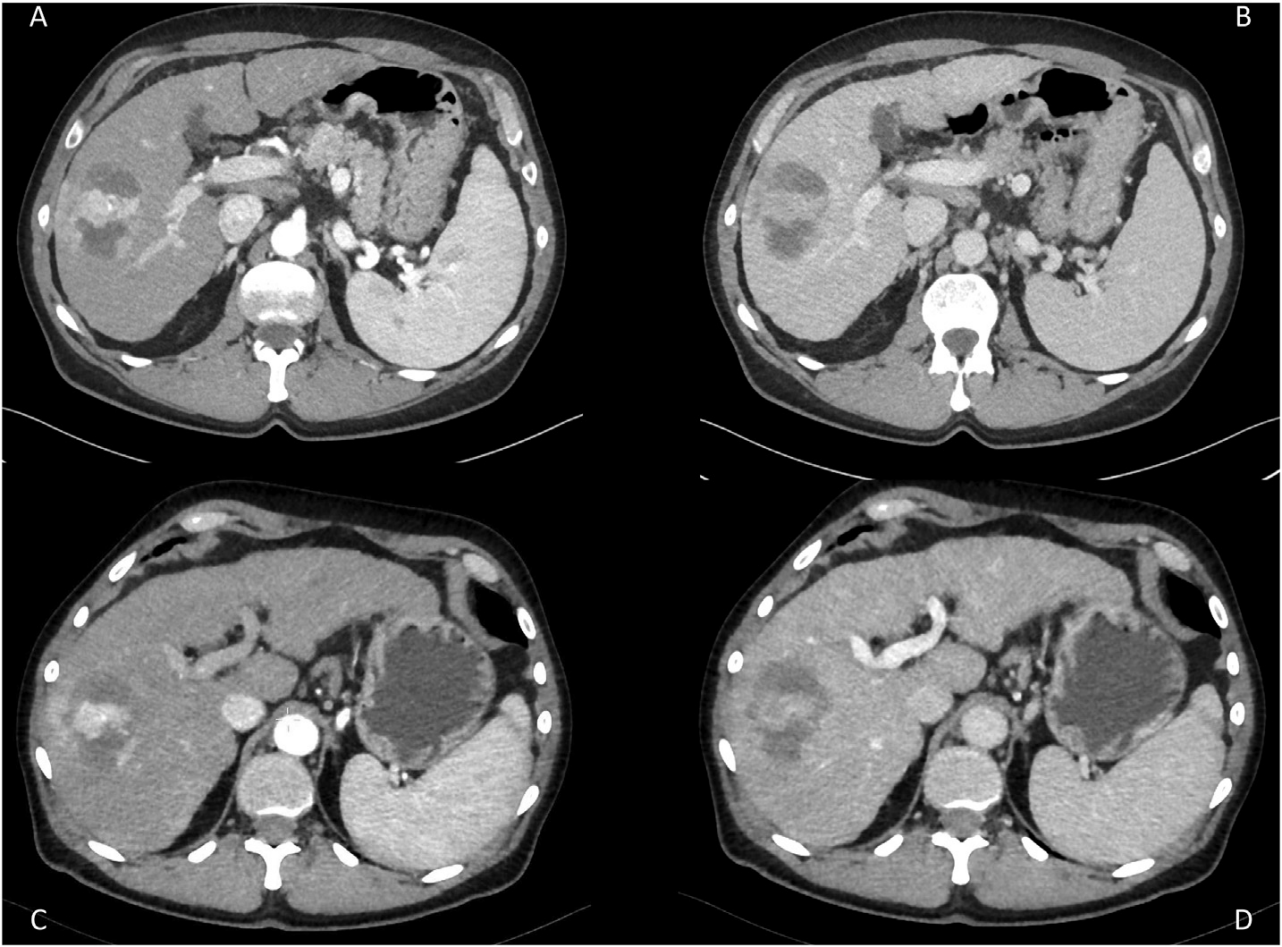
Contrast-enhanced cross-sectional images of abdominal 4-phase CT with A) arterial and B) PV phase and reconstructed images from CTP with C) A-CTP and D) PV-CTP phases.

Images were reconstructed using Advanced Modeled Iterative Reconstruction (ADMIRE)—an iterative postprocessing algorithm [16], a slice thickness of 3 mm, and convolution kernel of Bv40. Image stacks of the 4-phase CT and CTP scans were generated using ADMIRE 3 and 4, respectively (Figure 1). The latter ADMIRE grade was selected based on preliminary data showing that it yields an image quality roughly comparable to that of 4-phase scans with ADMIRE 3 reconstruction [16].

### 2.5 Reconstruction of arterial and PV phases in CTP

**Figure 2:**
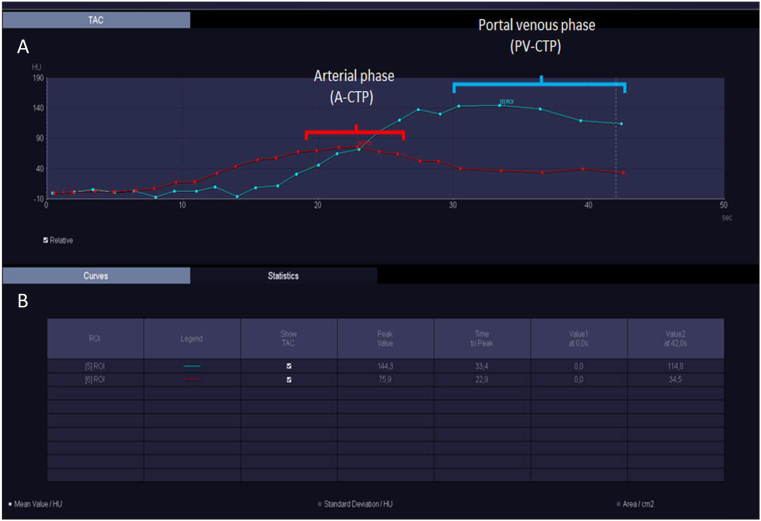
Images from syngo.via illustrating the CTP protocol. In total, 25 scans (dots on the curves) with variable temporal phases and enhancement of the arterial (red curve, ROI in the pancreas) and PV (blue curve, ROI in portal vein) phases were performed at different time points (row A). Peak times for the arterial and PV phases were measured (row B), and 5 stacks around the peak of the time attenuation curve of the pancreas for the arterial phase and the last 5 stacks for the PV phase were manually selected and merged to generate the A- and PV-CTP.

Syngo.via imaging software was used to extract the arterial and PV phases from CTP scans [14, 15]. To reconstruct A-CTP, 5 CTP image stacks representing the arterial phase were selected around the peak of the time attenuation curve of the pancreas—representing the maximum signal for the late arterial phase was measured. PV-CTP was reconstructed from the last 5 CTP image stacks (Figure 2). In both reconstructions, the 5 selected stacks were merged to create a new cross-sectional image set (Figure 1).

### 2.6 Quantitative image analysis

Quantitative image analysis was performed by determining image noise, signal-to-noise ratio (SNR), and contrast-to-noise ratio (CNR). A picture archiving and communication system workstation (IDS7; Sectra Imtec AB, Linköping, Sweden) was used to measure Hounsfield units (HU) and image noise. The SD of HU was used to calculate image noise and measurements were obtained by defining the ROI in left lobe of the liver, corpus of the pancreas, subcutaneous abdominal fat, lumbar paravertebral muscles, and abdominal aorta at the level of the diaphragm. The analysis was done by AM under supervision of MW. The SNR was calculated using Eq. (1) [15, 16].

(1) $$SNR\;of\;organ\;X=\frac{Mean\;HU\;of\;ROI\;of\;organ\;X}{SD\;of\;the\;ROI\;of\;organ\;X}$$

The CNRs of the abdominal aorta and main portal vein in arterial and PV phases were calculated using Eqs. (2) and (3).

(2) $$CNR_{aorta}=\frac{\left(HU_{aorta}-HU_{liver}\right)}{SD_{liver}}$$

(3) $$CNR_{main\;portal\;vein}=\frac{\left(HU_{main\;portal\;vein}-HU_{liver}\right)}{SD_{liver}}$$

### 2.7 Qualitative image analysis

Absolute assessment of image quality [17] was performed by 2 experienced radiologists (WB and MW, with 12 and 10 years of experience in abdominal radiology, respectively). Images were evaluated in 2 sessions separated by a 2-week interval in order to avoid recall bias. In each session, 13 A- and PV-CTP examinations and 13 4-phase CT examinations were randomly selected for assessment of subjective image quality and diagnostic performance. The former was evaluated according to the following criteria in the European guidelines for abdominal CT [18]: 1) visually sharp reproduction of the liver parenchyma; 2) visually sharp reproduction of pancreatic contours; 3) visually sharp reproduction of kidneys and proximal ureters; 4) visually sharp reproduction of lymph nodes <15 mm in diameter; 5) overall image quality for diagnostic purposes; 6) image noise; 7) image contrast; and 8) diagnostic confidence. These criteria were graded on a 5-point Likert scale (Table 1).

**Table 1:** 5-Point Likert-like scale used to assess subjective image quality and LI-RADS classification†.

Score	LI-RADS category
1: Excellent	1: Definitely benign
2: Good	2: Probably benign
3: Acceptable	3: Indeterminate
4: Suboptimal	4: Probably HCC
5: Poor	5: Definitely HCC

Abbreviations: HCC, hepatocellular carcinoma; LI-RADS, Liver Imaging Reporting and Data System.

† Image quality was compared between reconstructed computed tomography perfusion images and 4-phase examination; the scoring was performed by reviewers based on arterial phase enhancement (wash-in), portal venous washout, the existence of a capsule, lesion size, and threshold growth.

The criteria for diagnostic performance were as follows: number of lesions, position of lesions (Couinaud hepatic segment), size of lesions (mm), and LI-RADS category (version 2018) [19]. The reviewers independently evaluated each examination and a consensus reading of the image sets was used in the analysis.

### 2.8 Statistical analysis

Data for all participating patients were analyzed using SPSS v25 software (IBM, Armonk, NY, USA). Ordinal data were converted to numbers (1–5) and continuous data are presented as mean ± SD. Data were analyzed for normality with the Shapiro–Wilk test. A paired samples t-test was used to compare quantitative image quality measurements and performance diagnostics based on the number of detected lesions. Subjective image quality and diagnostic performance datasets were analyzed with the Wilcoxon signed-rank test. Cohen's kappa coefficient (κ) was used to calculate interobserver agreement in the number of detected lesions, with κ values ≤ 0.20, 0.21–0.40, 0.41–0.60, 0.61–0.80, and ≥0.81 corresponding to slight, poor, moderate, very good, and excellent agreement, respectively [20, 21]. P < 0.05 was considered statistically significant in all tests.

### 2.9 Ethical considerations

Ethics approval was obtained from the regional ethics and radiation protection committees (Dnr: 2016/43-31). Informed consent was obtained from each participant before the examinations.

## 3 Results

### 3.1 Quantitative image analysis

There were no significant statistical differences in image noise levels between A -CTP image sets and A 4-phase CT (all P > 0.05), with the exception of muscle tissue which had lower image noise in the 4-phase CT (P = 0.015) (Table 2). There were no significant differences in image noise between PV-CTP and PV 4-phase CT.

**Table 2:** Comparison of image quality parameters between reconstructed cross-sectional images from A- and PV-CTP phases and 4-phase CT images of A and PV phases (n = 26).

	A-CTP	A 4-phase CT	P value	PV-CTP	PV 4-phase	P value
**Image noise (HU)**						
Liver	11.69 ± 3.25	11.77 ± 1.88	0.91	11.88 ± 3.50	11.65 ± 1.55	0.73
Pancreas	16.81 ± 6.03	15,81 ± 3.68	0.40	13.96 ± 4.82	12.88 ± 2.72	0.32
Muscle	13.12 ± 3.73	11.23 ± 1.80	**0.015∗**	13.00 ± 4.24	11.42 ± 2.06	0.08
Fat	11.77 ± 4.60	10.19 ± 1.79	0.12	10.85 ± 2.96	10.38 ± 2.06	0.31
Aorta	13.54 ± 4.72	12.08 ± 1.79	0.10	11.77 ± 3.87	10.73 ± 1.56	0.17
**SNR**						
Liver	6.32 ± 1.61	6.88 ± 1.44	0.12	7.98 ± 2.68	8.87 ± 1.45	**0.027∗**
Pancreas	7.77 ± 2.85	7.77 ± 2.09	0.99	7.36 ± 2.86	6.87 ± 1.43	0.31
Muscle	4.63 ± 1.73	5.47 ± 1.30	**0.003∗**	4.85 ± 1.87	5.60 ± 1.33	**0.04∗**
Fat	12.78 ± 4.5	10.41 ± 2.06	**0.014∗∗**	13.22 ± 3.82	10.34 ± 2.33	**0.0001∗∗**
Aorta	20.80 ± 9.77	28.65 ± 8.70	**0.005∗**	13.19 ± 5.23	12.92 ± 2.98	0.80
**CNR**						
Aorta	17.24 ± 10.64	22.53 ± 8.43	0.059	4.50 ± 2.36	2.82 ± 0.95	**<0.001∗∗**
Main portal vein	3.85 ± 4.22	4.08 ± 2.87	0.63	7.54 ± 3.44	4.04 ± 0.96	**<0.001∗∗**

Data represent mean ± SD.

∗P < 0.05, 4-phase CT superior to A-/PV-CTP; ∗∗P < 0.05, A-/PV-CTP superior to 4-phase CT.

Abbreviations: A, arterial; A-CTP, arterial computed tomography perfusion; CNR, contrast-to-noise ratio; CT, computed tomography; PV, portal venous; PV-CTP, portal venous computed tomography perfusion; SNR, signal-to-noise ratio.

SNR showed no significant statistical difference between A-CTP and A 4-phase CT datasets for liver and pancreas (P > 0.05). The same was true for PV-CTP and PV 4-phase CT datasets for the pancreas and aorta (P > 0.05). SNR in 4-phase CT datasets of the muscle (arterial and PV), aorta (arterial), and liver (PV) were significantly higher than in the corresponding A- or PV-CTP datasets (all P < 0.05), but the opposite was true for SNR values of fat (all P < 0.05).

There were no significant differences in CNR between A-CTP and arterial 4-phase CT datasets (all P > 0.05); however, CNR values were significantly higher in PV-CTP than in PV 4-phase CT in the aorta and main portal vein (all P < 0.001).

### 3.2 Qualitative image analysis

#### 3.2.1 Subjective image analysis

There were significant differences between the 2 image reconstruction methods in the subjective quality of liver, pancreas, kidney, and lymph nodes as determined by image noise, image contrast, diagnostic confidence, and overall image quality in both arterial and PV phases (all P < 0.001) (Table 3), with 4-phase CT datasets having a higher subjective image quality than CTP datasets.

**Table 3:** Comparison of subjective image quality between A-/PV-CTP and 4-phase CT examinations of the upper abdomen†.

	A 4-phase CT	A-CTP	PV 4-phase CT	PV-CTP	P value
Image noise	2.15 ± 0.37	2.92 ± 0.85	2.15 ± 0.37	2.92 ± 0.85	*P* < 0.001
Image contrast	1.88 ± 0.65	2.65 ± 0.75	1.88 ± 0.65	2.65 ± 0.75	*P* < 0.001
Diagnostic confidence	1.31 ± 0.47	2.69 ± 0.88	1.31 ± 0.47	2.69 ± 0.88	*P* < 0.001
Overall image quality	1.92 ± 0.48	2.88 ± 0.86	1.92 ± 0.48	2.88 ± 0.86	*P* < 0.001
Visualization of liver	2.00 ± 0.40	3.00 ± 0.75	2.00 ± 0.40	3.00 ± 0.75	*P* < 0.001
Visualization of pancreas	2.00 ± 0.28	2.81 ± 0.85	2.00 ± 0.28	2.81 ± 0.85	*P* < 0.001
Visualization of kidney and ureters	1.96 ± 0.34	2.73 ± 0.83	1.96 ± 0.34	2.73 ± 0.83	*P* < 0.001
Visualization of lymph nodes	2.00 ± 0.28	2.81 ± 0.90	2.00 ± 0.28	2.81 ± 0.90	*P* < 0.001

Data represent mean ± SD.

Abbreviations: A, arterial; A-CTP, arterial computed tomography perfusion; CT, computed tomography; PV, portal venous; PV-CTP, portal venous computed tomography perfusion.

† Note that arterial and portal venous phases of each examination had the same Likert score.

#### 3.2.2 Diagnostic performance

**Figure 3:**
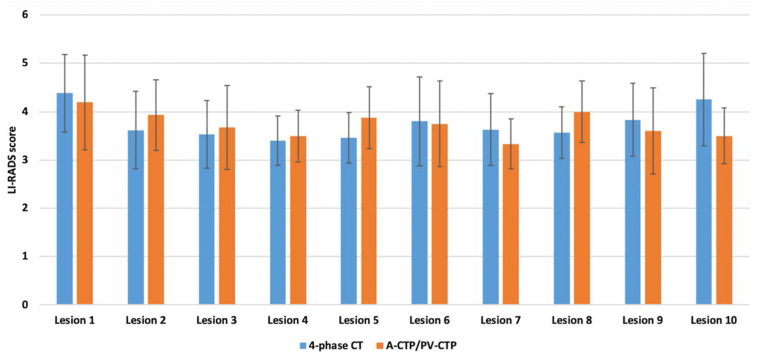
Comparison of LI-RADS classification categories (1–5) between 4-phase CT and reconstructed images of CTP (A-/PV-CTP) in examinations of the upper abdomen in up to 10 detected lesions per patient. X: number of detected lesions; Y: mean LI-RADS categories.

There were no statistical significant differences in the mean total number of detected lesions, position of lesions, lesion size, and LI-RADS category between 4-phase CT and A-/PV-CTP images (all P > 0.05) (Table 4). But more lesions overall and especially more LI-RADS grade 4 and 5 lesions were detected in 4-phase CT datasets (Table 5). There were several LI-RADS category 3 (n = 12), category 4 (n = 9), and category 5 (n = 3) lesions that were undetected or categorized lower by CTP. Both the 4-phase CT and CTP datasets showed reproducibility in per lesion assessment of detected HCC lesions according to LI-RADS criteria (Figure 3).

**Table 4:** Comparison of diagnostic performance between 4-phase CT and reconstructed CTP datasets (A-/PV-CTP).

	4-phase CT	A-/PV-CTP	P value
Mean number of detected lesions	4.73 ± 3.45	3.81 ± 3.85	0.073
Mean number of lesions in right lobe	3.42 ± 1.40	2.73 ± 1.6	>0.05
Mean umber of lesions in left lobe	1.31 ± 1.94	1.08 ± 1.11	>0.05
Mean LI-RADS category (1–5)	3.75 ± 0.33	3.74 ± 0.27	>0.05
Mean lesion size (mm)	20.01 ± 11.47	19.13 ± 12.29	>0.05
Data represent mean ± SD.			

	A/PV 4-phase CT	A/PV-CTP
Total number of detected lesions (n)	123	99
Number of lesions in right lobe (n)	89	71
Number of lesions in left lobe (n)	34	28
**Number of lesions based on weigh**		
Number of lesions weight (50–70 kg	35	37
Number of lesions weight (71–90 kg	34	28
Number of lesions weight (>90 kg)	54	34

Abbreviations: A-CTP, arterial computed tomography perfusion; CT, computed tomography; LI-RADS, Liver Imaging Reporting and Data System; PV-CTP, portal venous computed tomography perfusion.

**Table 5:** Comparison of total number of lesions detected in 4-phase CT and A-/PV-CTP phases based on LI-RADS category.

	LI-RADS 2	LI-RADS 3	LI-RADS 4	LI-RADS 5	Total
4-phase CT	1	53	42	27	123
CTP (A-/PV-CTP	1	41	33	24	99
Number of missed lesions in A-/PV-CTP (%		22.6%	21.4%	11.1%	19,5%

Abbreviations: A-CTP, arterial computed tomography perfusion; CT, computed tomography; CTP, computed tomography perfusion; LI-RADS, Liver Imaging Reporting and Data System; PV-CTP, portal venous computed tomography perfusion.

Relationship between number of detected lesions in 4-phase CT and reconstructed CTP images of the liver and patient weight and abdominal diameter.

**Figure 4:**
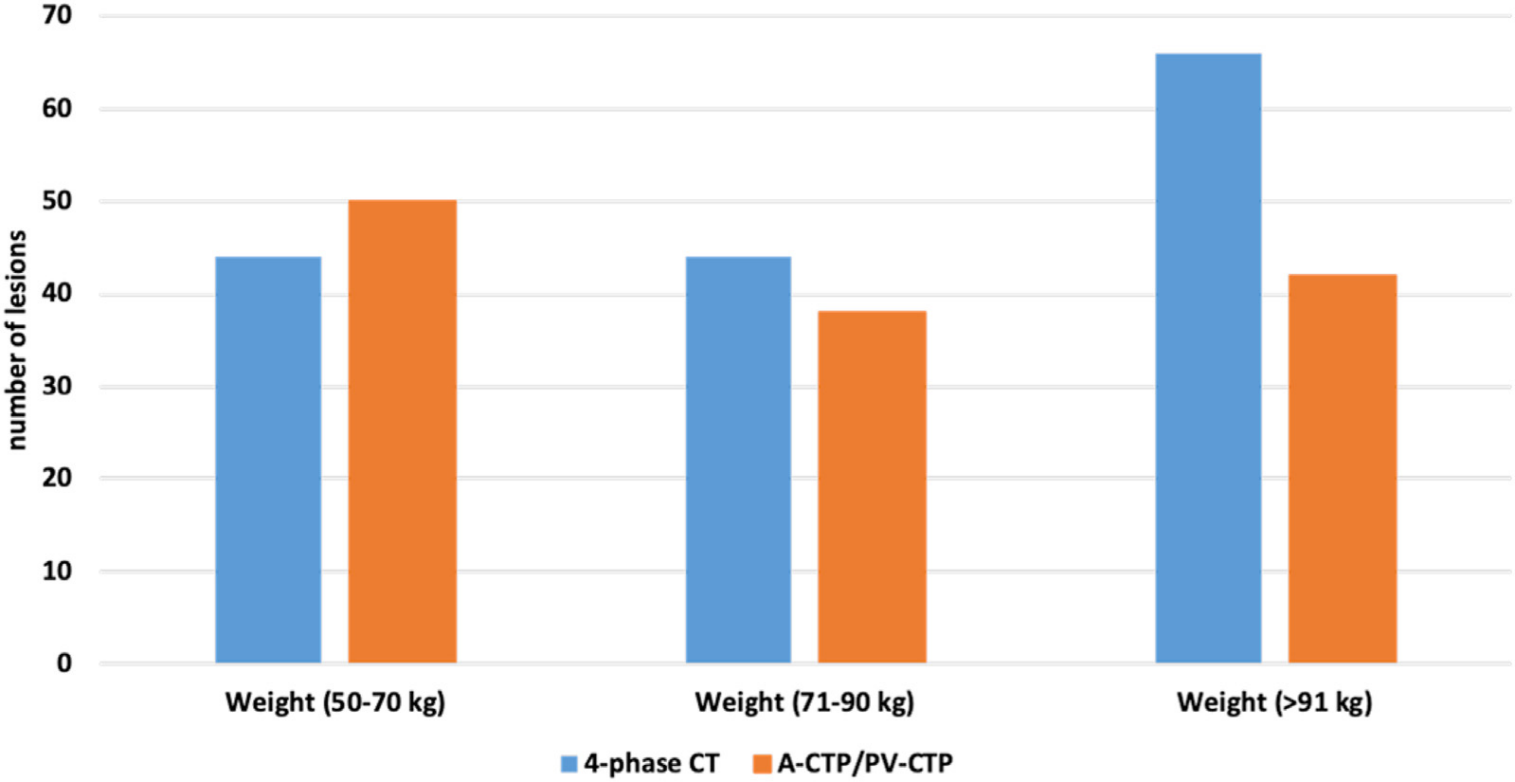
Relationship between patient weight and total number of detected lesions in a 4-phase CT and reconstructed images of A- and PV-CTP phases in examinations of the upper abdomen.

**Figure 5:**
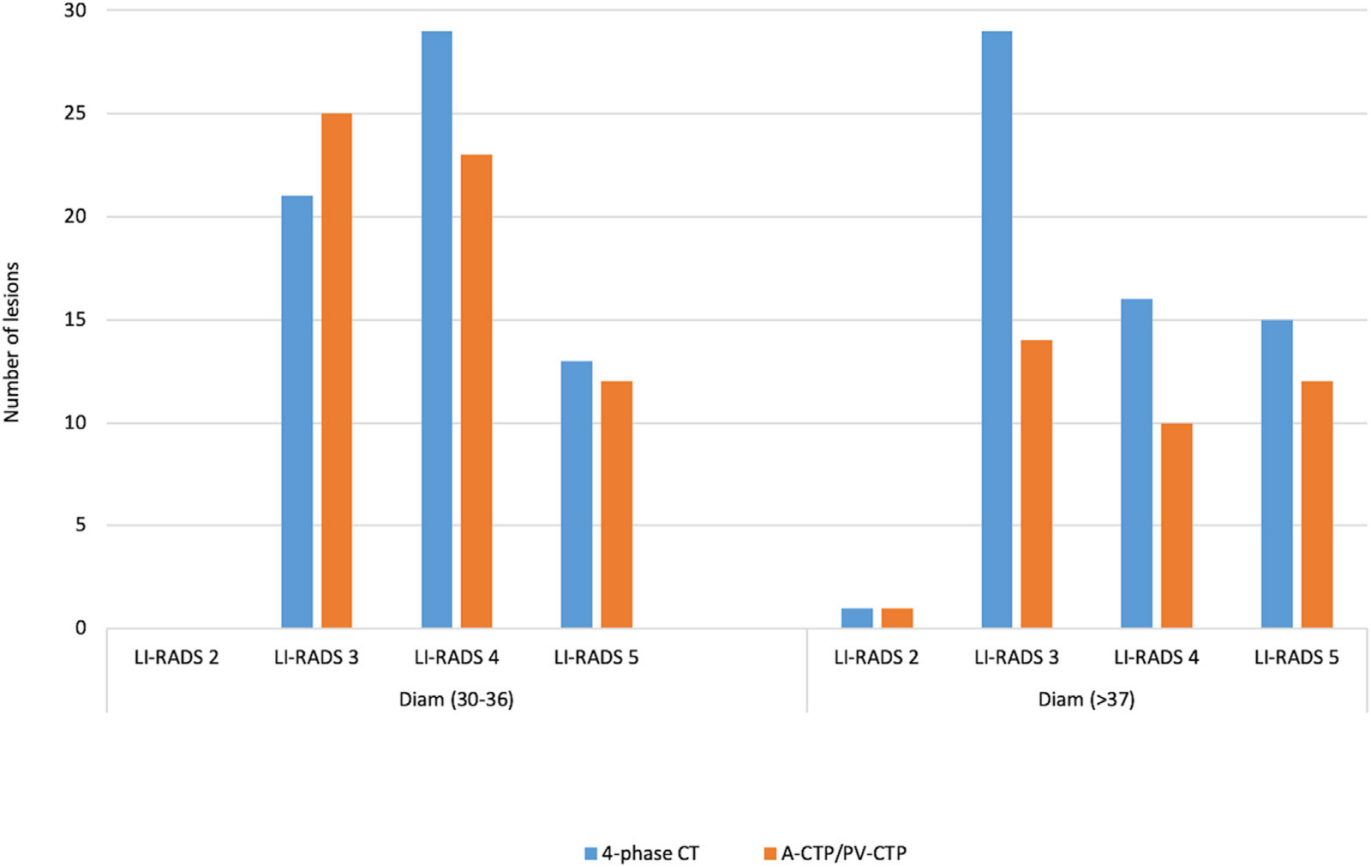
Relationship between the total number of detected lesions in 4-phase CT and reconstructed CTP images of the upper abdomen and patient's abdominal diameter and LI-RADS categories. Diam: diameter in cm.

There was a clear relationship between body weight as well as diameter and the number of lesions detected in 4-phase CT and A-/PV-CTP datasets. Compared to 4-phase CT, fewer lesions were detected in A-/PV-CTP images in heavier patients (>90 kg). Additionally, more lesions were detected in 4-phase CT images compared to A-/PV-CTP images in patients with an abdominal diameter >37 cm (Figure 4). These lesions were in all 3 relevant LI-RADS categories (ie, 3, 4, and 5) (Figure 5). The undetected lesions were not evenly distributed across the patient population: after stratifying patients by body weight and abdominal diameter, the number was lower in patients weighing >90 kg and/or with an abdominal diameter >37 cm.

#### 3.2.3 Interobserver agreement

Cohen's κ test showed very good (κ = 0.62, P = 0.001) and moderate (κ = 0.45, P = 0.001) concordance between the number of lesions detected by the 2 radiologists in 4-phase CT and A-/PV-CTP images, respectively.

#### 3.2.4 Radiation dose

The mean effective dose (±SD) in 4-phase CT examinations was 26.6 ± 12.89 mSv (range: 11.9–58.1 mSv). The average effective dose of the arterial and PV scans was 12.5 ± 6.05 mSv (range: 5.8–28.8 mSv). The mean effective dose in total (±SD) in CTP examinations was 17.5 ± 3.26 mSv (range: 13.0–24.8 mSv). Thus, an approximately 30% lower radiation dose was delivered with the CTP protocol compared to 4-phase CT. The mean effective dose was calculated by multiplying the dose length product (DLP) values of the respective scans by 0,015 (k conversion coefficient of the abdomen) in accordance to Christner et al. [22].

## 4 Discussion

CTP can overcome some limitations of multiphase CT in the monitoring of tumor behavior over time and assessment of therapeutic response by providing quantitative parameters in the image analysis [1, 2, 14]. For CTP to be accepted in routine practice by radiologists and clinicians, CTP image quality must be improved so that it is comparable to that of the current gold-standard methods (4-phase CT and multiparametric MRI). As mentioned earlier, the image quality of CTP is generally lower than that of 4-phase CT because of the lower radiation dose delivered at each time point and the fact that most CTP examinations still are reconstructed by filtered back projection. Other disadvantages of CTP are the sheer bulk of a scan—with thousands of images that can be time-consuming to read—as well as an increased risk of overdiagnosis because only the quantitative images are evaluated [14, 15]. In order to address these problems, we investigated whether CTP image series reconstructed with iterative kernels were comparable to those obtained by 4-phase CT in terms of quality and diagnostic performance and could thus replace the latter, with the advantage of providing potentially useful quantitative information when needed.

Results from the quantitative image analysis (image noise, SNR, and CNR) showed that reconstructed CTP datasets (ie, A- and PV-CTP) were equivalent to 4-phase CT datasets in most of the examined tissues. Regarding diagnostic performance, the reconstructed CTP images had adequate quality, with the result that per-lesion LI-RADS classifications of detected HCC lesions were the same using both imaging methods. However, the analysis of subjective image quality showed that the reconstructed CTP datasets were inferior to those obtained by 4-phase CT. A possible explanation for this is the use of a lower tube voltage in the CTP protocol to reduce the radiation dose. Moreover, the comparable number of lesions detected with the 2 methods indicated similar levels of sensitivity, provided that the patient weight was <90 kg and abdominal diameter was ≤37 cm on a mean lesion detection basis. However, in addition several LI-RADS 4 and LI-RADS 5 lesions were not detected or misclassified by CTP. This would not be acceptable in a clinical setting as lesions with LI-RADS 4 and 5 either have a high or absolute indication for treatment.

There were lesions detected by 4-phase CT that were not observed in the reconstructed CTP images. These lesions were in all 3 relevant LI-RADS categories (ie, 3, 4, and 5) and would warrant either close follow-up or treatment depending on the grade. The undetected lesions were not evenly distributed across the patient population: after stratifying patients by body weight and abdominal diameter, the number was lower in patients weighing >90 kg and/or with an abdominal diameter >37 cm. It is possible that these lesions were missed because a fixed tube voltage of 70 kVp in the CTP protocol was used in all examinations regardless of patient weight. In general, a higher voltage is required for adequate tissue penetration in obese patients to reduce noise and achieve good image quality [23]. With increasing patient diameter and weight, the penetration of radiation with 70 kVp compared to 120 kVp will decrease drastically, which increases the amount of noise and lowers the signal to noise ratio. Our results suggest that CTP is not a viable alternative for patients in this group [24].

Our findings on image quality of CTP are partly consistent with previous reports [14, 15]. But Wang et al. [15] did not observe statistically significant differences between 3D reconstructed images from CTP and standard multiphase CT in HCC patients, and concluded that CTP can replace the 4-phase CT examination. However, we found significant differences between the 2 imaging methods in several respects that may be attributable to differences in protocol acquisition parameters, reconstruction algorithms, and CT technique. One discrepancy is the radiation dose of the linearly blended 120-kVp protocol used by Wang et al., which was 5.6 mSv in total compared to 12.5 mSv in our study and may have reduced the quality of their images (which is supported by the lower noise and higher SNR and CNR values in our study), resulting in an overestimation of image quality of their mean temporal datasets. Another difference is that we reconstructed our temporal datasets with ADMIRE 4, whereas Wang et al. and Fischer et al. [14] used filtered back projection. Several studies have demonstrated that iterative ADMIRE reconstructions yield less noise and higher CNR [24, 25]. This is supported by our results, in which mean temporal arterial and PV scans generally showed lower noise levels and higher SNR and CNR values.

In summary, our temporal datasets were comparable and the reason for the apparent inferiority of CTP to 4-phase CT examinations may be the higher radiation doses in our reference study.

A strength of the present study was the application of LI-RADS classifications to the detected lesions, which is essential in clinical HCC assessment as LI-RADS is the standard system of communication between radiologists and referring physicians [19]. Other comparable studies did not use LI-RADS classifications, precluding an evaluation of the clinical impact of different study protocols [14, 15].

The CTP imaging method has several advantages. Firstly, arterial and PV phase image reconstructions improved the image quality and diagnostic performance of CTP. Secondly, the CTP protocol allowed an approximately 60% reduction in iodine contrast (from 118 to 50 ml), thereby reducing the risk of contrast-induced nephropathy. Thirdly, the radiation dose was also decreased compared to 4-phase CT, minimizing the radiation-related cancer risk to patients. Finally, the CTP protocol enabled quantitative evaluation of tumors, which is useful for differentiating lesions and predicting treatment outcomes [8, 10, 26, 27].

There were several limitations to the present study that may have influenced our conclusions. Firstly, the sample size was small. Secondly, a scan time of 45 s (contrast delay ca 52 s) in CTP examinations may not always correspond to the PV phase of 4-phase CT with a 60-s delay. On the other hand, the majority of reconstructed images showed adequate contrast enhancement in the PV phase of CTP and after ca 32 s of contrast delay a plateau can be seen in the HU units in the liver. Thirdly, in the CTP image acquisition protocol, tube voltage was not adjusted based on body weight, which may have resulted in a lower image quality for obese patients.

## 5 Conclusions

In conclusion, our results show that mean temporal images of arterial and PV phases reconstructed from low-dose CTP datasets had comparable quantitative image quality to those obtained with the standard method (4-phase CT). Moreover, the rate of detection of lesions and their LI-RADS classifications were similar with the 2 methods (for patients weighing <90kg), although CTP datasets had inferior subjective image quality and our results suggest that CTP might be able to be used instead of 4-phase CT for the diagnosis of HCC in patients weighing <90 kg or with an abdominal diameter <37 cm but should not be used in the present protocol form for patients weighing >90 kg or with an abdominal diameter of >37 cm as a significant amount of LI-RADS 4 and LI-RADS 5 lesions were missed/miscategorized in the latter patient group.

## Declarations

### Author contribution statement

Mischa Woisetschläger: Conceived and designed the experiments; Performed the experiments; Analyzed and interpreted the data; Contributed reagents, materials, analysis tools or data; Wrote the paper.

Aref Mohammadi: Performed the experiments; Analyzed and interpreted the data; Contributed reagents, materials, analysis tools or data; Wrote the paper.

Wolf Bartholomae: Performed the experiments; Analyzed and interpreted the data; Wrote the paper.

### Funding statement

This work was supported by the County Council of Östergötland, Sweden (LFoU).

### Data availability statement

Data will be made available on request.

### Declaration of interests statement

The authors declare no conflict of interest.

### Additional information

No additional information is available for this paper.

## References

[1] Haj-Mirzaian A., Kadivar A., Kamel I.R., Zaheer A. (2020). Updates on imaging of liver tumors. Curr. Oncol. Rep..

[2] Navin P.J., Venkatesh S.K. (2019). Hepatocellular carcinoma: state of the art imaging and recent advances. J. Clin. Transl. Hepatol..

[3] Marrero J.A., Kulik L.M., Sirlin C.B., Zhu A.X., Finn R.S., Abecassis M.M., Roberts L.R., Heimbach J.K. (2018). Diagnosis, staging, and management of hepatocellular carcinoma: 2018 practice guidance by the American association for the study of liver diseases. Hepatology.

[4] Galle P.R., Forner A., Llovet J.M., Mazzaferro V., Piscaglia F., Raoul J.L., Schirmacher P., Vilgrain V. (2018). EASL clinical practice guidelines: management of hepatocellular carcinoma. J. Hepatol..

[5] Adibi A., Shahbazi A. (2014). Automatic bolus tracking versus fixed time-delay technique in biphasic multidetector computed tomography of the abdomen. Iran. J. Radiol..

[6] Bae K.T. (2010). Intravenous contrast medium administration and scan timing at CT: considerations and approaches. Radiology.

[7] Rengo M., Bellini D., De Cecco C.N., Osimani M., Vecchietti F., Caruso D., Maceroni M.M., Lucchesi P., Iafrate F., Palombo E., Paolantonio P., Ferrari R., Laghi A. (2011). The optimal contrast media policy in CT of the liver. Part II: clinical protocols. Acta Radiol..

[8] Kim S.H., Kamaya A., Willmann J.K. (2014). CT perfusion of the liver: principles and applications in oncology. Radiology.

[9] Voizard N., Cerny M., Assad A., Billiard J.S., Olivié D., Perreault P., Kielar A., Do R.K.G., Yokoo T., Sirlin C.B., Tang A. (2019). Assessment of hepatocellular carcinoma treatment response with LI-RADS: a pictorial review. Insights Imaging.

[10] Hatem Shalaby M., Ali Shehata K.A. (2017). CT perfusion in hepatocellular carcinoma: is it reliable?, Egypt. J. Radiol. Nucl. Med..

[11] Goh V., Halligan S., Daley F., Wellsted D.M., Guenther T., Bartram C.I. (2008). Colorectal tumor vascularity: quantitative assessment with multidetector CT--do tumor perfusion measurements reflect angiogenesis?. Radiology.

[12] Hayashi M., Matsui O., Ueda K., Kawamori Y., Kadoya M., Yoshikawa J., Gabata T., Takashima T., Nonomura A., Nakanuma Y. (1999). Correlation between the blood supply and grade of malignancy of hepatocellular nodules associated with liver cirrhosis: evaluation by CT during intraarterial injection of contrast medium. AJR Am. J. Roentgenol..

[13] Fischer M.A., Marquez H.P., Gordic S., Leidner B., Klotz E., Aspelin P., Alkadhi H., Brismar T.B. (2017). Arterio-portal shunts in the cirrhotic liver: perfusion computed tomography for distinction of arterialized pseudolesions from hepatocellular carcinoma. Eur. Radiol..

[14] Fischer M.A., Leidner B., Kartalis N., Svensson A., Aspelin P., Albiin N., Brismar T.B. (2014). Time-resolved computed tomography of the liver: retrospective, multi-phase image reconstruction derived from volumetric perfusion imaging. Eur. Radiol..

[15] Wang X., Henzler T., Gawlitza J., Diehl S., Wilhelm T., Schoenberg S.O., Jin Z.Y., Xue H.D., Smakic A. (2016). Image quality of mean temporal arterial and mean temporal portal venous phase images calculated from low dose dynamic volume perfusion CT datasets in patients with hepatocellular carcinoma and pancreatic cancer. Eur. J. Radiol..

[16] Woisetschläger M., Henriksson L., Bartholomae W., Gasslander T., Björnsson B., Sandström P. (2020). Iterative reconstruction algorithm improves the image quality without affecting quantitative measurements of computed tomography perfusion in the upper abdomen. Eur. J. Radiol. Open..

[17] Kataria B., Nilsson Althen J., Smedby O., Persson A., Sokjer H., Sandborg M. (2019). Image quality and pathology assessment in CT Urography: when is the low- dose series sufficient?. BMC Med. Imag..

[18] Office for Official Publications of the European Communities (1996).

[19] Chernyak V., Fowler K.J., Kamaya A., Kielar A.Z., Elsayes K.M., Bashir M.R., Kono Y., Do R.K., Mitchell D.G., Singal A.G., Tang A., Sirlin C.B. (2018). Liver imaging reporting and data system (LI-RADS) version 2018: imaging of hepatocellular carcinoma in at-risk patients. Radiology.

[20] McHugh M.L. (2012). Interrater reliability: the kappa statistic. Biochem. Med..

[21] Koo T.K., Li M.Y. (2016). A guideline of selecting and reporting intraclass correlation coefficients for reliability research. J. Chiropr. Med..

[22] Christner J.A., Kofler J.M., McCollough C.H. (2010). Estimating effective dose for ct using dose-length product compared with using organ doses: consequences of adopting international commission on radiological protection publication 103 or dual-energy scanning. Am. J. Roentgenol..

[23] Guimaraes L.S., Fletcher J.G., Harmsen W.S., Yu L., Siddiki H., Melton Z., Huprich J.E., Hough D., Hartman R., McCollough C.H. (2010). Appropriate patient selection at abdominal dual-energy CT using 80 kV: relationship between patient size, image noise, and image quality. Radiology.

[24] Kalra M.K., Woisetschläger M., Dahlström N., Singh S., Lindblom M., Choy G., Quick P., Schmidt B., Sedlmair M., Blake M.A., Persson A. (2012). Radiation dose reduction with sinogram affirmed iterative reconstruction technique for abdominal computed tomography. J. Comput. Assist. Tomogr..

[25] Kataria B., Althé J.N., Smedby Ö., Persson A., Sökjer H., Sandborg M. (2018). Assessment of image quality in abdominal CT: potential dose reduction with model-based iterative reconstruction. Eur. Radiol..

[26] Hamdy A., Ichikawa Y., Toyomasu Y., Nagata M., Nagasawa N., Nomoto Y., Sami H., Sakuma H. (2019). Perfusion CT to assess response to neoadjuvant chemotherapy and radiation therapy in pancreatic ductal adenocarcinoma: initial experience. Radiology.

[27] Tamandl D., Waneck F., Sieghart W., Unterhumer S., Kölblinger C., Baltzer P., Ba-Ssalamah A., Loewe C. (2017). Early response evaluation using CT-perfusion one day after transarterial chemoembolization for HCC predicts treatment response and long-term disease control. Eur. J. Radiol..

